# Generic prescription drug price increases: which products will be affected by proposed anti-gouging legislation?

**DOI:** 10.1186/s40545-018-0156-8

**Published:** 2018-11-21

**Authors:** Rena M. Conti, Kevin H. Nguyen, Meredith B. Rosenthal

**Affiliations:** 1 Department of Markets, Public Policy, and Law, Institute for Health System Innovation and Policy, Questrom School of Business, Boston University, 595 Commonwealth Avenue, Office 514B, Boston, MA 02215 USA; 2 Department of Health Policy and Management, Harvard T.H Chan School of Public Health, Boston, MA USA

**Keywords:** Prescription drugs, Price competition, Generic drugs, Price increases, Costs, Legislation

## Abstract

**Background:**

In the United States (U.S.), large price increases for selected generic drugs have elicited public outrage. Recent legislative proposals aim to increase price transparency and identify outlier drug “price spikes.” It is unknown how many and what types of products would be highlighted by such efforts.

**Methods:**

IQVIA Health Incorporated’s National Sales Perspectives™ provided sales, use and price data for all generic prescription products (unique molecule-manufacturer-formulation combinations) sold in the U.S. We estimated annual prescription price levels and changes between 2013 and 2014. We identify drugs with annual prescription price increases in excess of the medical consumer price index (CPI), and in excess of 15% or 20%, per legislative proposals. We reported annualized inflation-adjusted mean, standard deviation (SD), median, and 95th percentile prescription price increases and percentage of products exceeding the growth in the medical CPI. We fitted logistic regression models to identify characteristics of drugs associated with each category of price increase.

**Results:**

We analyzed data for 6,182 generic products. The mean inflation-adjusted price increase among all generic products was 38% (SD 1,053%), the median, 2%; the 95th percentile, 135%; and the mean price level, $29.69 (SD $378.44). Approximately half of all products experienced price increases in excess of the growth in the medical CPI; 28% had price increases greater than 15% and 23% had price increases greater than 20%. Drugs exceeding outlier thresholds exhibited lower baseline price levels than the mean price level observed among all generic drugs. The most consistent characteristic predicting whether a product would exceed “price spike” thresholds proposed in legislation is the being supplied by only one manufacturer.

**Conclusions:**

“Price spikes” among generic drugs in 2014 were more common than newspaper stories and legislative hearings suggest. While the cross-sectional association between an indicator of being sold by only a single manufacturer and the probability of meeting specific price growth thresholds is suggestive of an economically intuitive causal story, future work should delve more deeply into whether decreases in generic competition explain the dramatic price increases that have captured the public’s attention in recent years.

## Background

Some pharmaceutical companies and their executives are now household names in the United States (U.S.) thanks to publicity around their pricing practices [1–3]. In the U.S. the market for “off-patent” generic drugs is typically portrayed thus: demand for generics is strong since many are central to chronic disease treatment and entry among suppliers and subsequent price competition is vigorous, thereby placing downward pressure on pricing levels and trends. However, as the lay press and published studies have documented, there have been massive price increases for “off-patent” generics that tend to be the standard of care in selected diseases, such as the antibiotic doxycycline [4–6]. Concern has also focused attention on price increases among generics that have few therapeutic competitors and may enjoy some market exclusivity [6, 7], such as Pyrimethamine (Daraprim™^)^ used to treat toxoplasmosis and cystoisosporiasis and the epinephrine auto-injector (EpiPen™) used to treat anaphylaxis [8]. Indeed, in 2012–2013, the prices of some generics increased so much that they appear to have impacted national prescription drug spending [9–12]. These trends have ignited public fury [13].

Public outcry has generated a corresponding flurry of activity by politicians [14]. 2017 started out with President Trump tweeting that the “artificially high price of drugs” must be brought down “immediately.” [15] Acting on recommendations from an investigation by the U.S. Senate Committee on Aging, key legislators in the U.S. Congress introduced two federal bills in 2017: [16] A U.S. Senate bill sponsored by Senators Franken and Klobuchar proposed increased transparency and routine assessments of drug price trends [17] and a bill sponsored by the late Senator McCain and Senator Baldwin proposed similar remedies [18]. To date, neither have progressed out of committee and remain unenacted [19].

The states have been more successful in pursuing legislative action to address escalating drug prices [20]. California [21], Louisiana [22], New York, Nevada and Vermont [23] have enacted price transparency laws, while Maryland [24], has passed legislation requiring drug price transparency and defined explicit thresholds for identifying drugs exhibiting “price spikes,” similar to the proposed Senate legislation. Oregon’s legislature passed a similar drug transparency and price gouging bill in February 2018 (https://www.statesmanjournal.com/story/news/politics/2018/02/28/prescription-drug-price-transparency-bill-passes-oregon-house/382665002/).

The sheer volume of legislative proposals and the increasing likelihood of passage of these bills in 2017 and 2018 is new [14], although there was substantive legislative activity on the topic of drug pricing dating back to 2009 [25] related to a number of high profile drug shortages and corresponding price increases were reported by consumer advocates and the U.S. Food and Drug Administration (FDA) [26, 27]. In the first 12 weeks of 2018 alone, 53 drug pricing transparency bills were introduced in 23 state legislatures [28].

All of this activity begs a simple empirical question: How common are massive price increases among generic drugs and for which drugs do we see price increases significant enough to meet price-gouging thresholds set by current legislative proposals? Only three studies we are aware of have examined generic pricing trends, all of which have used subsets of national data and focused exclusively on oral generic drugs dispensed through retail pharmacies. The Government Accountability Office (GAO) analyzed Medicare Part D IQVIA data and found that from early 2010 to mid-2015 more than 20% of generic drugs had undergone reimbursement price increases of over 100%. The Centers for Medicare and Medicaid Services (CMS) analyzed prescriptions reimbursed under Medicaid fee for service (FFS) between July 1, 2013 and June 30, 2014 and measured pricing trends using data from the National Average Drug Acquisition Cost pricing files that measure drug acquisition costs for retail pharmacies. They found that generic drugs with very significant acquisition cost increases (more than 100%) accounted for approximately 9% of $6.0 billion in total Medicaid expenditures on generic drugs over the study period. Expanding the definition of a significant increase in acquisition costs to include increases of more than 20% means that less than 15% of Medicaid expenditures on generic drugs experienced a significant increase. In a 2015 report investigating the Medicaid drug rebate program, the HHS Office of the Inspector General (OIG) examined price increases in generic prescription drugs with the highest Medicaid reimbursement rates between 2005 and 2014. OIG identified 869 drugs that were in the top 200 Medicaid generic drugs at least once in the duration, and assessed whether they exceeded a statutory inflation factor, the average manufacturing price (AMP). They found that on average 22% of generic prescription drugs had an actual quarterly AMP that exceeded the inflation-adjusted AMP over the study period, ranging from 18% in 2006 to 35% in 2014.

In this study, we used the leading source of national data on prices, use, and spending for every prescription drug available in the U.S. between 2013 and 2014. To put the previous studies into wider context, our sample includes oral, infused, injected or otherwise formulated generic drugs dispensed through all channels and covered by insurer pharmacy and medical benefits. Our study objectives were to examine generic drug prescription price levels and changes in this period; to operationalize currently proposed price gouging definitions; and to identify drug characteristics associated with meeting outlier thresholds defined by legislation.

## Methods

### Data

We obtained quarterly national data on the quantities and wholesale dollar sales of all prescription drugs approved for sale in the U.S. from IQVIA Health Incorporated’s National Sales Perspectives™ (NSP) database for 2013 and 2014. NSP data derive from a projected audit describing 100% of the national unit volume and dollar sales in every major class of trade and distribution channel for U.S. prescription pharmaceuticals. The sample is based on over 1.5 billion annual wholesale transactions and does not include free samples of prescription drugs.

NSP provides information on specific chemical and branded names. The data also include the name of drug’s “labeler,” which is the U.S. FDA’s terminology for the owner of the New Drug Application (used for approval of a new drug before commercialization) or the Abbreviated New Drug Application (used for approval of an off-patent generic drug) in the Orange Book. NSP reports “extended units” measuring the number of single items (such as a bottle or a packet of tablets or capsules) contained in a unit or shipping package purchased by providers and pharmacies at the molecule-quarter-year level. NSP also reports “dollar sales” defined as the amount all channels pay for the molecule-quarter-year across all dispensed prescriptions. Dollar sales for each molecule-quarter-year were converted into 2016 U.S. dollars using the Consumer Price Index (CPI) all urban inflation calculator [29].

NSP includes the patent status of each molecule, a variable denoting whether each drug is “branded,” “generic,” or “branded generic”. “Branded generics” are non-originator drugs that fall into one of the following categories: novel dosage forms of off-patent products, often in combination with another molecule; on patent with a trade name, but a molecule copy of an originator product; off patent with a trade name; or off patent without a trade name and from a single source or co-licensed [30]. We included “generic” drugs in our sample, defined as those in the generic and branded generic categories. Vaccines, over-the-counter products, and vitamins were excluded from the analytic sample.

We calculated the inflation adjusted price of one extended unit sold of a drug by quarter-year by dividing the inflation-adjusted drug sales by drug extended units sold in each quarter-year. The resulting mean inflation-adjusted prescription prices reflect the actual invoice prices pharmacies, hospitals and clinics pay for a unit of the drug, whether purchased directly from a manufacturer or indirectly via a wholesaler or chain warehouse.

NSP data also include a number of other drug characteristics related to their demand (formulation, a 16 category modified measure of anatomic therapeutic class, blockbuster sales status, and whether the drug received FDA approval for at least one orphan indication) and indicators of limited supply (count of manufacturers) (see Appendix 1).

### Identification of outlier Price change thresholds

Using a Lexis Nexus search, keywords “prescription” and “price” and “outlier” and “legislation” proposed or enacted between January 1, 2015 and July 1, 2017, we identified and reviewed state and federal legislation seeking to monitor prescription drug prices and identify drug price increase outliers. Table 4 in Appendix 2 enumerates a sample of identified state and federal legislative proposals intended to moderate generic price increases. All focus on wholesale acquisition costs (so-called “WAC”) and its changes as the measure of drug price to be monitored. This choice aligns well with the measure of price per prescription we estimate using NSP data, since both ignore most rebates and discounts that might be obtained by pharmacy benefit managers, group purchasing organizations and other direct purchasers [31].

The proposals differ with respect to the basic elements of the analysis, including the price increase thresholds defining a “spike” and time periods in which drugs are assessed for outlier price changes. The Vermont law defines a drug prescription price spike as one seeing increases of more than 50% in their WAC over five years. The Maryland bill requires the state attorney general to be notified of off-patent drug prescription price increases that meet or exceed 50% in their WAC over one year or more in a given year but does not establish a minimum threshold for the definition of price gouging. The Senate bill sponsored by Franken and Klobuchar defines price spikes as annual increases above the medical CPI, with even higher penalties for increases that exceed 15% and 20% annually. The legislation also contemplates an alternative calculation of penalties based on 5-year growth as compared to compounded versions of the annual thresholds matching the benchmark used by a 2016 study by the U.S. GAO reporting that from early 2010 to mid-2015 more than 20% of generic drugs had undergone price increases of over 100% [32]. Because it offers a range of thresholds that bracket most of the other proposed “price spike” definitions, we use the Frank and Klobuchar penalty thresholds to simulate the number and types of products that would be affected if such legislation were adopted.

### Statistical analysis

In our analysis, we define a product as a unique combination of molecule, seller, and formulation. First, we estimated and report annualized inflation-adjusted means and standard deviations for annual price changes for all generic products between 2013 and 2014. Second, we applied the Franken-Klobuchar price spike thresholds to drugs in these eras to report the number of drugs exceeding the penalty thresholds. To place these outliers into context, we also estimated and report their mean prescription price levels and utilization per quarter. We take this approach because the same percentage increase will have a bigger impact on patients and payers when the baseline price is higher. The proposed Maryland legislation, for example, suggests increased scrutiny for drugs that cost more than $80 per 30-day course. Third, we describe the distribution of product characteristics that might be associated with price increases and also report mean prices for subgroups of products. Finally, we estimated logistic regression models to identify drug characteristics associated with exceeding price spike thresholds. For each regression, the dependent variable was an indicator variable for whether the product met or exceeded the price spike threshold. Independent variables included the product characteristics listed in the Appendix 1. We report odds ratios and interpret those with *p*-values < 0.05 to be statistically significant. All analyses used STATA 14.0 (College Station, Texas). The Institutional Review Board of the University of Chicago deemed this study not human subjects research.

## Results

In our analytic sample there were 2,285 distinct molecules and 6,182 unique molecule-seller-formulation combinations for which price data exist from both 2013 and 2014. Three hundred and seventy-eight manufacturers supplied these products to the U.S. market in during this period.

In 2013–2014 the mean inflation-adjusted price increase among all generic products was 38% (SD 1,053%), the median was 2%, the 95th percentile was 135% and the mean price level was $29.69 (SD $378.44) (Table 1). There were 3,102 products (1,648 molecules) that exceeded the medical CPI growth threshold (50% of products), manufactured by 319 manufacturers (84% of all manufacturers have at least one product that met this threshold). These products had a mean inflation-adjusted price increase of 93% (SD 1,485%); with the median and 95th percentile price increase of 17% and 249%, respectively. The mean price level for products exceeding the medical CPI growth threshold was $43.35 (SD $521.16). There were 1,713 products that exceeded the 15% price increase threshold (28% of products), manufactured by 213 manufacturers (56% of manufacturers). These products had a mean inflation-adjusted price increase of 162% (SD 1,995%); with the median and 95th percentile price increase of 41% and 376%, respectively. The mean price level of products exceeding 15% price growth was $30.72 (SD $385.12). There were 1,425 products that met the 20% threshold (23% of products), manufactured by 197 manufacturers (52% of manufacturers). These products had a mean inflation-adjusted price increase of 191% (SD 2,187%); with the median and 95th percentile price increase of 52% and 424%, respectively. The mean price level of products exceeding 20% price growth was $22.63 (SD $292.56).

**Table 1:** Drug Price Increase “Hot Spots”^a^

Price Change Distribution, Measured at the Drug Level	2013-2014			
	Overall	% > Medical CPI	% > 15%	% > 20%
Number of Unique Molecules in Sample	2,285	1,648	987	826
Molecule-Seller-Formulation Combinations in Sample	6,182	3,102	1,713	1,425
Percentage of Molecule-Seller-Formulation Combinations Meeting Threshold	-	50%	28%	13%
Number of Manufacturers in Sample	378	319	213	197
Percentage of total manufacturers producing molecules meeting threshold	-	84%	56%	52%
Quarterly Use (millions of units)				
Mean	7.83	7.03	7.49	7.94
Standard Deviation	3.48	3.67	2.62	2.76
Quarterly Sales (millions of dollars)				
Average	3.66	3.78	3.53	2.83
Standard Deviation	1.73	2.01	1.71	1.13
Price Change				
Mean	38%	93%	162%	191%
Median	2%	17%	41%	52%
95th Percentile	135%	249%	376%	424%
Standard Deviation	1053%	1485%	1995%	2187%
Price Level, 2013 ($)				
Average	29.69	43.35	30.72	22.63
Standard Deviation	378.44	521.16	385.12	292.56
Hot Spot Market Share^b^				
Based on Total Extended Units Sold	-	45%	27%	23%
Based on Total Inflation-Adjusted Sales	-	25%	12%	8%

^a^Authors’ calculations using IQVIA Health NSP data. All analyses used STATA 14.0 (College Station, Texas)

^b^Hot Spot market share was calculated in terms of total extended units (total extended units sold by hot spot products divided by total extend units sold of all products) and total inflation-adjusted sales (total inflation-adjusted sales of products by hot spot products divided by total inflation-adjusted sales of all products) by era

Products exceeding the medical CPI threshold for 2013–2014 generated an average of 7.03 million unit sales (SD 3.67 million) and $3.78 million in dollar sales (SD $2.01 million). Products exceeding the 15% threshold generated 7.49 million in unit sales (SD 2.62 million) and $3.53 million in quarterly sales (SD $1.71 million). Products exceeding the 20% threshold generated 7.94 million in unit sales (SD 2.76 million) and $2.83 million in quarterly sales (SD $1.13 million).

Table 2 describes the distribution of product characteristics and average price levels in 2013. Overall 27% of products were supplied by only one manufacturer while more than half (53%) were supplied by 5 or more manufacturers. Average prices were more than 35 times higher for products supplied by a single manufacturer than those supplied by five or more manufacturers. Sixty-four percent of all products were supplied in oral formulations; 14% were injectable, and 22% were in some other form (e.g. topical). Injectable products were by far the most expensive per unit while orals were the least expensive (mean price $133.76 vs. $2.63). Branded generic products, which were more than 13 times as expensive than unbranded generics, made up 21% of all generic products. Approximately 10% of products sold fewer than 1,000 units in a quarter in 2013 (in the quarter preceding the price change measurement) and these low volume products had prices more than 8 times higher than other products. The majority (79%) of products had sales less than $100 million while 1% of products had sales in excess of 1 billion dollars. About 8% of all products were designated as orphan products and these products had prices more than 15 times higher than non-orphan products.

**Table 2:** Product characteristics and average price levels^a^

Product characteristic	Percent of products (*N* = 6,182)	Mean (SD) USD Price Level in 2013
Manufacturer Count per Product		
1	27%	92.90 (721.02)
2	6%	23.47 (108.27)
3	8%	16.13 (71.54)
4	6%	8.79 (37.44)
≥ 5	53%	2.65 (16.94)
Formulation		
All Others	22%	41.23 (526.71)
Injectable	14%	133.76 (753.03)
Oral	64%	2.63 (14.24)
Patent Status		
Branded Generic	21%	112.03 (822.57)
Generic	79%	8.36 (54.11)
Volume in previous period		
Very low volume (<1,000 units per quarter)	10%	142.14 (1,077.18)
All other drugs	90%	17.65 (186.61)
Orphan Drug Designation		
No	92%	13.53 (78.45)
Yes	8%	214.16 (1,293.70)

^a^Authors’ calculations using IQVIA Health NSP data. All analyses used STATA 14.0 (College Station, Texas)

Table 3 summarizes the results of the logistic regressions. Compared to all generic products, those exceeding the medical CPI threshold in 2013–2014 had higher odds of having only one or two manufacturers (OR 1.53, 1.26, *p* < 0.01, *p* = 0.04), lower odds of being a non-branded generic (OR 0.49, p < 0.01), and higher odds of having low volume (OR 1.28, *p* = 0.02). Compared to all generic products, those exceeding the 15% threshold in 2013–2014 had higher odds of having only one or two manufacturers (OR 1.33, 1.31, *p* = 0.02, *p* = 0.03), and higher odds of having low volume (OR 1.38, *p* < 0.01). Compared to all generic products, those exceeding the 20% threshold in 2013–2014 had higher odds of having only one manufacturer (OR 1.30, *p* = 0.05) or three manufacturers (OR 1.35, *p* = 0.01), and higher odds of having low volume (OR 1.46, *p* < 0.01).

**Table 3:** Odds ratios from regression analysis of factors associated with price increases above thresholds

	2013-2014					
	% > Medical CPI		% > 15%		% > 20%	
	Odds Ratio	*p*-value	Odds Ratio	*p*-value	Odds Ratio	*p*-value
Manufacturer count per product						
1	1.53	<0.01	1.33	0.02	1.33	0.03
2	1.26	0.04	1.31	0.03	1.07	0.60
3	1.05	0.64	1.10	0.11	1.35	0.01
4	0.93	0.48	1.10	0.42	1.11	0.42
≥ 5	Ref	-	Ref	-	Ref	-
Formulation						
All Others	Ref	-	Ref	-	Ref	-
Injectable	0.88	0.30	0.90	0.46	0.97	0.85
Oral	0.83	0.10	1.02	0.90	1.11	0.39
Patent Status						
Branded Generic	Ref	-	Ref	-	Ref	-
Generic	0.49	<0.01	0.81	0.09	0.97	0.84
Volume in previous period						
Volume >1,000 units	Ref	-	Ref	-	Ref	-
Volume <1,000 units	1.28	0.02	1.38	<0.01	1.46	<0.01
Orphan Drug Designation						
No	Ref	-	Ref	-	Ref	-
Yes	1.15	0.15	1.04	0.71	1.12	0.31

## Discussion

Generic prescription drug prices have been escalating rapidly. In a single year, 2014, the average increase in generic prices was 38%, reflective of extremely high price increases for some products with a median price increase just above that of the medical CPI (approximately 2%). This price growth is much higher relative to inflation in other medical treatments: between 2008 and 2012 inpatient care prices increased at a mean annual rate of 1.8% [33] while primary care physician salaries increased between 3.5 and 5.5% [34]. Moreover, these prices affect a large share of the population. In contrast to many consumer goods and specific medical treatments, generic prescription drugs are widely used by the general public and chronically consumed [35, 36].

Our results also suggest that 13% of products exhibited annual growth in excess of 20%, the highest penalty threshold in the Franken-Klobuchar bill. This suggests “price spikes” among generic drugs are much more common than newspaper stories and legislative hearings focused on a handful of “bad actor” manufacturers of selected products may suggest. Irrespective of threshold definition, products exceeding outlier thresholds exhibited lower levels in price than the mean price level observed among all generic products. Outliers also exhibited lower mean quarterly use levels and sales compared to all generic products. These results suggest that the absolute impact of these price increases on payer and patient’s cost sharing budgets may be less than the prevalence estimates suggest. Combined, these results underscore the wisdom of Maryland’s legislation providing discretion to the state’s attorney general in investigating price spikes, particularly for drugs with price levels of $80 per prescription or more.

The number of manufacturers consistently appeared to be associated with higher price increases. These results suggest inelastic demand and/or limited supply may play a role in driving individual manufacturers to pursue significant price increases [26, 37]. This is a surprising result and suggests that the much-lauded success of the Hatch-Waxman Act of 1984, which aimed to promote vigorous generic entry and price competition, may not ensure sustained price competition among generic drug manufacturers over time [38]. More empirical work is needed to examine the potential for limited entry, merger and acquisition activity or subsequent exit among generic drug manufacturers to substantially increase prices [39]. Unfortunately, U.S. federal policy has only limited experience and modest success in introducing more competition once the structure of product markets has evolved to become a monopoly or limited oligopoly [40, 41]. Despite recent federal legislation introduction like the Franken-Klobuchar bill, as well as the U.S. FDA commissioner Gottlieb’s announcement of the agency’s proactive review of manufacturer applications into generic drugs with limited suppliers, there is little precedent and much uncertainty associated with using tools at the disposal of the FDA to increase supply of generic drugs in the U.S.

## Conclusion

As policy makers consider the path forward, the potential for unintended consequences should be carefully assessed. Notably, the use of publicly assessed thresholds to define outlier price increases, like those suggested in the Frank-Klobuchar bill, might paradoxically lead to more widespread price increases among generic drugs. Here, the use of a publicly defined floor to invoke increased policy maker scrutiny may be interpreted by manufacturers as defining a ceiling for product price increases over defined periods of time that may be implemented without triggering public scrutiny. Consequently, policy makers should assess whether the main benefits of making drug prices public – i.e. taming price increases through the naming and shaming of bad actors – may be better served by keeping monitoring efforts private. Indeed, the state of Maryland has chosen this alternative path to reduce the potential for unintended consequences.

There are a number of limitations to this analysis. First, while NSP are the most comprehensive source of aggregate prescription drug use and spending, they are based on a subset of years. Future work should extend this analysis through more recent years. Second, we chose to focus on this analysis on generic drugs since inclusion of branded drugs would complicate the interpretation of results. Future study should examine price changes among branded drugs. Third, this study is descriptive and further study is required to empirically evaluate the causes of observed price increases.

Pharmaceutical policy in the U.S. attempts to balance the need to provide adequate returns on investment against ensuring access to prescription drugs at affordable prices. Rising prices for existing drugs, including many generic drugs that have long since lost their marketing exclusivity in recent years, presents new challenges. The trends and correlates we describe in generic drug pricing suggest that current policies promoting competition between drugs and manufacturers to maintain access and affordability may need to be revisited and ultimately strengthened. Despite the promise of these polices they are not without potential unintended consequences.

## Appendix 1

**Definitions**

- Count of manufacturers was constructed by aggregating drugs by chemical name and counting unique labelers in each quarter-year. We then counted the annual number of unique manufacturers for each molecule.
- Formulation: NSP data for each NDC provides formulation codes (part of the NDC code) to classify drugs into several categories: oral solid tablets or capsules (“oral”); injectable or infusible products (“injectable”); topical preparations; inhaled products, and “other” formulations (e.g. ocular drugs and patches) (“other”).
- Product: We defined product as a unique combination of molecule, seller, and formulation.
- Patent status: Generic products are categorized based on whether they are marketed using a proprietary name (branded generic) or only the chemical name.
- Low volume: We defined low volume to be fewer than 1,000 units per quarter in the period before me measure the price change (the bottom decile of volumes).
- Orphan is a designation granted by the Food and Drug Administration for drugs to treat rare conditions. This designation is requested by a sponsor.
- Therapeutic class: NSP data for each molecule contains World Health Organization’s 244 four-digit anatomic therapeutic classification (ATCs), following IQVIA’s annual reports. We report results using an aggregated classification system of the 16 two-digit ATCs relating to the general target of biological activity, such as “cardiovascular” or “antineoplastic and immunomodulating.” [Note: results for therapeutic class dummy variables not shown]

## Appendix 2

**Table 4:** Proposed legislation

Title	Summary	Year Proposed
An Act to promote transparency and cost control of pharmaceutical drug prices (Massachusetts)^a^	Would require the development of a list of critical prescription drugs of substantial public interest. For each drug listed, manufacturers would report economic expenditures from research and development to marketing and advertising. This data would then be compiled into a report that would inform the public on prescription drug prices and their role in overall health care spending in the commonwealth. Report could include recommendations for actions to lower prescription drug costs and spending across the commonwealth while maintaining access to quality health care.	2015
Improving Access to Affordable Prescription Drugs Act (Franken Klobuchar Bill)^b^	Expands reporting requirements for drug manufacturers and establishes corresponding civil penalties for noncompliance. Adds reporting requirements for certain nonprofit patient-assistance programs. Requires the Government Accountability Office (GAO) to report to Congress on the impact of patient-assistance programs on prescription-drug pricing and expenditures. Requires the Centers for Medicare and Medicaid Services (CMS) to negotiate prices for certain prescription drugs under the Medicare program. Requires Centers for Medicare and Medicaid Innovation to test specified models for negotiating drug prices. Establishes an excise tax on prescription drugs subject to price spikes. Lessens prescription-drug cost sharing requirements under qualified health plans, group health plans, and the Medicare program. Requires drug manufacturers to provide drug rebates for drugs dispensed to certain low-income individuals under the Medicare program.	2017
Prescription Drug and Health Improvement Act of 2017 (Franken Reed Brown Gillibrand)^c^	Would require the Secretary of Health and Human Services to negotiate lower covered part D drug prices on behalf of Medicare beneficiaries.	2017
Fair Accountability and Innovative Research Drug Pricing Act of 2017 (McCain Baldwin Price Bill)^d^	Would require reporting on the justification for drug price increases and for other purposes.	2017
An act relative to prescription drugs (Vermont)^e^	In an attempt to increase transparency, typically the first step towards cost containment, this bill would require The Green Mountain Care Board, in collaboration with the Department of Vermont Health Access, to identify annually up to 15 prescription drugs on which the State spends significant health care dollars and for which the wholesale acquisition cost has increased by 50 percent or more over the past five years or by 15 percent or more over the past 12 months. For each drug identified, the Office of the Attorney General shall require the manufacturer to provide justification for the increase in the wholesale acquisition cost increase.	2016
Pharmaceutical Cost Transparency Act of 2016 (California)^f^	This bill would require each manufacturer of a prescription drug made available in California that has a wholesale acquisition costs of $10,000 or more annually or per course of treatment to file a report, no later than May 1 of each year, with the Office of Statewide Health Planning and Development on the costs of each qualifying drug.	2015
An act to require manufacturers of pharmaceutical drugs to report cost and utilization information (North Carolina)^g^	Would require an annual report of drug costs and use that can be used by policymakers, government agencies and others to understand pharmacy cost trend in an attempt to make information available to the public about the cost and utilization of pharmaceutical drugs made available in North Carolina.	2015

^a^ https://malegislature.gov/Bills/189/Senate/S1048

^b^ https://www.congress.gov/115/bills/s771/BILLS-115s771is.pdf

^c^ https://www.congress.gov/bill/115th-congress/senate-bill/348

^d^ https://www.gpo.gov/fdsys/pkg/BILLS-115s1131is/pdf/BILLS-115s1131is.pdf

^e^ https://legislature.vermont.gov/assets/Documents/2016/Docs/ACTS/ACT165/ACT165%20As%20Enacted.pdf

^f^ https://leginfo.legislature.ca.gov/faces/billNavClient.xhtml?bill_id=201520160AB463

^g^ https://webservices.ncleg.net/ViewBillDocument/2015/3341/0/DRH10298-MM-102

## Abbreviations

AMP: Average manufacturing price
ATC: Anatomical therapeutic classification
CMS: Centers for medicare and medicaid services
CPI: Consumer price index
EpiPen™: Epinephrine auto-injector
FDA: Food and Drug Administration
FFS: Fee for service
GAO: Government Accountability Office
HHS: Health and Human Services
NDC: National Drug Code
NSP: National Sales Perspectives™
OIG: HHS Office of the Inspector General
SD: Standard deviation
U.S.: United States
WAC: Wholesale acquisition costs
